# Acute Appendicitis After Spine Fusion for Adolescent Idiopathic Scoliosis: A Case Report

**DOI:** 10.7759/cureus.3522

**Published:** 2018-10-30

**Authors:** Kessiena L Aya, David Yngve, Richy M Charls

**Affiliations:** 1 Department of Orthopaedic Surgery and Rehabilitation, The University of Texas Medical Branch, Galveston, USA

**Keywords:** acute appendicitis, adolescent idiopathic scoliosis, postoperative pain management, posterior spinal fusion

## Abstract

Appendicitis is a common cause of pediatric abdominal pain, largely occurring in the second decade of life. We present the case of a 14-year-old girl who underwent an uncomplicated posterior spinal fusion with instrumentation for scoliosis, who later developed abdominal pain, nausea, and emesis secondary to acute appendicitis. Her hospital course was significant for prolonged intravenous use of narcotics for pain control and subsequent constipation but negative for abdominal pain or tenderness during her admission. While gastrointestinal complications are a common cause of unplanned 30-day readmissions in the pediatric population, appendicitis has yet to be reported. To our knowledge, this is the first case report of acute appendicitis after posterior spinal fusion, likely resulting from postoperative pain management.

## Introduction

Acute appendicitis is a life-threatening condition and is the most common condition leading to emergent abdominal surgery in the pediatric population [1]. The underlying pathology of this condition is generally due to lymphoid hyperplasia secondary to conditions, such as inflammatory bowel disease, infections, fecal stasis, and/or fecalith obstruction [2]. Established postoperative complications of posterior spinal fusion (PSF) include surgical site infections, pseudoparalysis, respiratory complications, excessive blood loss, venous thromboembolism, and implant-related complications [3-4]. While gastrointestinal complications are a notable cause of unplanned 30-day readmission in pediatric patients following posterior spinal fusion, acute pancreatitis, emesis, superior mesenteric syndrome, constipation, and dehydration were the most common gastrointestinal issues reported in most studies [4-5]. We present a case of acute appendicitis occurring after a posterior spinal fusion and review the data to date on abdominal pain after spine surgery. To the best of our knowledge, acute appendicitis after posterior spinal fusion for adolescent idiopathic scoliosis (AIS) has not been reported elsewhere in the literature.

## Case presentation

The patient and guardian were informed that data concerning this case would be submitted for publication, and consent was obtained. Institutional Review Board approval was not required in accordance with our institution’s policies.

**Figure 1:**
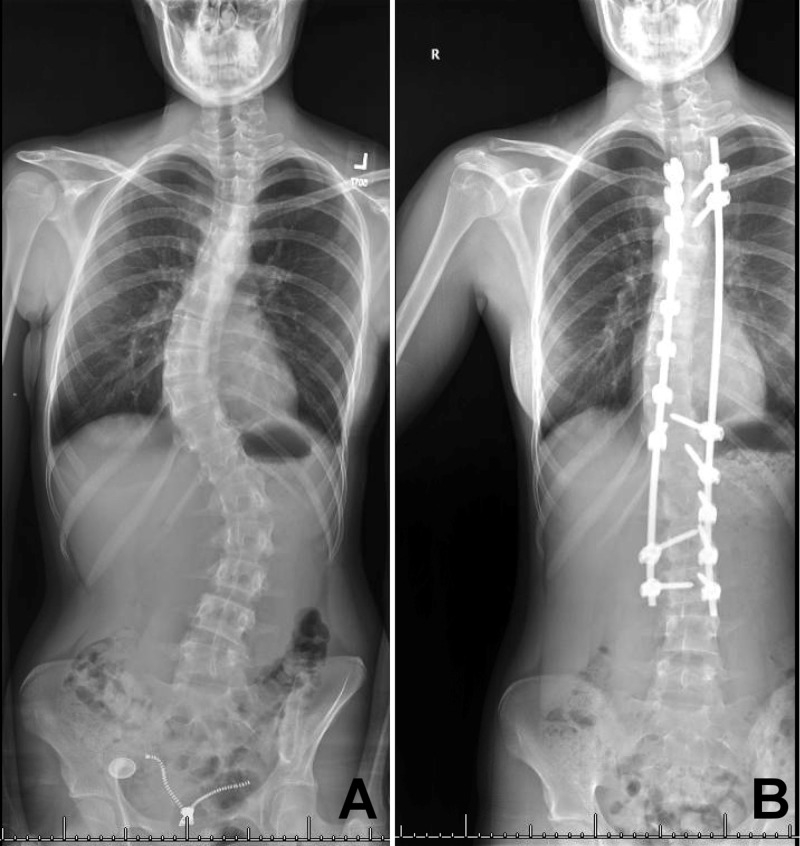
Preoperative (A) and postoperative (B) anteroposterior scoliosis radiographs

A 14-year-old girl presented to our clinic with a seven-month history of progressive low back pain without any neurologic deficits. She had been diagnosed with AIS by her pediatrician at age 12 and was being monitored for progression. Her past medical and surgical history was significant for an umbilical hernia repair at age five and one episode of self-resolving, non-specific abdominal pain five months prior to her spine surgery. On her physical exam, she had a noticeable right thoracic prominence on forward bending. Radiological examination demonstrated a 53-degree right convex thoracic curve and a 49-degree left convex lumbar curve (Figure 1A). She was Risser Stage 4 (United States version). Of note, radiographs also demonstrated findings of an enlarged liver. Bracing was not indicated. Given the magnitude of the curve and her level of skeletal maturity, the patient opted for surgical intervention.

She subsequently underwent segmental posterior spinal instrumentation and fusion from T4 to L3. The free-hand technique was used to place pedicle screws bilaterally at T4, T5, T11, L2, and L3; left-sided screws were placed at T6 through T10, and right-sided screws were placed at T12 and L1. All the screws were checked with fluoroscopy after insertion with anteroposterior and lateral views (Figure 1B).

While her surgery was uneventful, the patient was noted to require more than the standard protocol of pain medication for adequate control postoperatively. She was subsequently discharged on postoperative day six, which is two to three days longer than the average course of our typical adolescent scoliosis patients. Apart from her prolonged need for intravenous narcotics, she also had approximately five days of constipation that resolved prior to discharge. The rest of her hospital stay was routine; she was out of bed on postoperative day 1, walked around the unit on postoperative day 2, and was tolerating solid food on postoperative day 3.

The patient was subsequently discharged home with a routine course of multimodal pain medication and a bowel regimen. At that time, she had no complaints of abdominal pain nor tenderness to abdominal palpation. At her follow-up clinic visit on postoperative day 17, the patient was healing routinely and had no significant complaints or concerns. Radiographs taken in the office demonstrated posterior spinal instrumentation without evidence of hardware complication.

**Figure 2:**
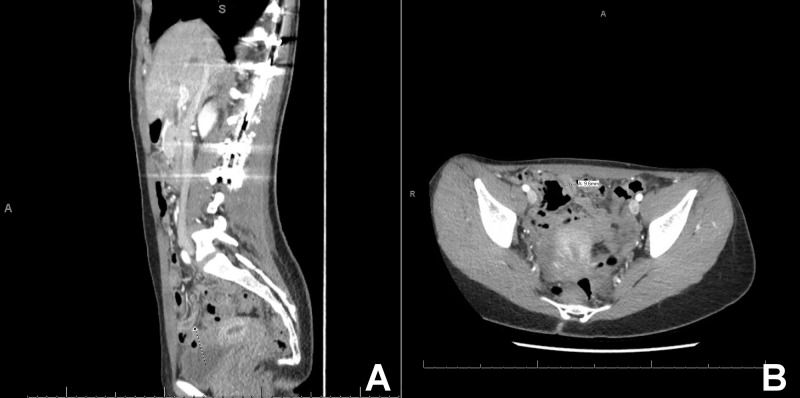
Contrast-enhanced computed tomography (CT) scan Select sagittal (A) and axial (B) contrast-enhanced CT images demonstrating a hyperenhancing thick-walled tubular structure in the right lower quadrant/pelvis, likely representing an inflamed appendix

On postoperative day 19, the patient presented to the emergency room with a 24-hour history of right lower quadrant pain and vomiting. Per her mother, the patient had been on pain medications since the surgery but recently began to wean off them. On physical exam, she had tenderness to palpation in the right lower quadrant, guarding, and a positive Rovsing’s sign. All of these findings were highly suggestive of acute appendicitis. Laboratory results showed a negative pregnancy test, leukocytosis, and elevated liver blood tests. A stat computed tomography scan revealed a dilated appendix with enhancing walls concerning for acute appendicitis (Figure 2A, 2B). A pediatric colorectal surgeon was consulted. After formally diagnosing acute appendicitis, she then underwent an emergent laparoscopic appendectomy. Gross tissue examination by pathology revealed an intact vermiform appendix with scant distal mesoappendix. The patient had healed routinely by her six-month follow-up. To date, she has experienced no recurrence of abdominal symptoms.

## Discussion

In a retrospective cohort study, Martin et al. concluded that gastrointestinal (GI) issues were the most common causes for readmission among pediatric patients after scoliosis surgery. Based on their findings, up to 15.4% of pediatric readmissions were due to GI issues [4]. While acute pancreatitis, superior mesenteric syndrome, constipation, and dehydration were the most common etiologies of 30-day unplanned readmission in their patient population, idiopathic abdominal pain and ileus were not uncommon. Therefore, the clinical diagnosis of postoperative appendicitis is complicated, as the abdominal pain may be attributed to a host of other expected postoperative GI issues.

Acute appendicitis is an inflammatory process involving the appendix, a blind-ended tube connected to the cecum. The appendix base is usually located 2 cm from the ileocecal valve, although there are a number of variable anatomic locations. Appendicitis normally presents in children and adolescents as vague abdominal pain, low-grade fever, nausea, vomiting, and diarrhea, with the majority of patients having tenderness at McBurney’s point (a point over the right side of the abdomen that is two-thirds of the space from the umbilicus to the anterior superior iliac spine) [6]. However, the absence of specific historical or physical exam findings does not necessarily rule out the disease [7].

Despite the prevalence of acute appendicitis, it is an uncommon GI complication following pediatric spinal deformity surgery. In a multicenter retrospective cohort study, Freedman et al. found that acute appendicitis accounted for 34.1% of all cases of severe constipation necessitating an emergency room visit in patients less than 18 years of age if the diagnosis was missed initially [8]. Severe constipation was also strongly correlated to narcotic usage for pain control (adjusted odds ratio (aOR) of 2.63). This was a likely cause of our patient’s GI complaints, as her postoperative hospital course was complicated by prolonged intravenous narcotics, which resulted in an extended period of constipation. The delayed presentation of this complication could be associated with the use of routine postoperative antibiotics [9]. In addition, the patient’s requirement for laxatives to alleviate her constipation increased her risk of appendiceal rupture.

Numerous measures have been explored with regard to reducing general GI complications after surgery. An example of an effective reduction of GI complications includes the usage of epidural analgesia instead of opioid narcotics for pain relief, as this can prevent paralytic ileus and facilitate proper gut motility [9]. In considering how to reduce constipation-induced appendicitis after spinal surgery in our patient, preoperative bowel preparation as compared to no bowel preparation before spinal surgery was shown to have no statistical advantage, as shown by Olsen et al. [10]. However, a case report highlighting the use of alvimopan, a mu-opioid receptor antagonist, produced a rapid return of proper bowel function following a two-stage posterior spinal fusion [11]. By alleviating bowel paralysis to allow appropriate fecal transit, the risk of fecalith obstruction leading to appendicitis would be reduced [12]; thus, the use of alvimopan as a prophylactic agent against GI complications, including appendicitis, for patients undergoing spinal procedures should be further investigated for high-risk patients.

Several treatment modalities are available in the management of acute appendicitis. Open appendectomy has traditionally been the mainstay treatment for this condition, but with the advent of laparoscopic techniques, this is decreasingly being utilized. Although an open appendectomy is associated with less cost and operative time overall [12], the laparoscopic approach was shown to have a decreased rate of short- and long-term postoperative complications, such as surgical site infection, with the disadvantage of increased risk for development of an intra-abdominal abscess [10]. In consideration of these factors, our patient underwent a laparoscopic appendectomy for her acute appendicitis.

## Conclusions

Spinal fusion surgery to correct deformity is a fairly safe and effective procedure for patients with adolescent idiopathic scoliosis. Gastrointestinal complications after surgery should be well understood by all parties involved, including the pediatric spine surgeon, patient, and caregivers. Understanding the potential development of these complications allows physicians to plan ahead by utilizing various prophylactic measures to ensure proper bowel function. Prevention of these complications can reduce patient morbidity and mortality, as well as reduce the costs of readmission.
